# Engineered single nucleotide polymorphisms in the mosquito MEK docking site alter *Plasmodium berghei* development in *Anopheles gambiae*

**DOI:** 10.1186/1756-3305-7-287

**Published:** 2014-06-23

**Authors:** Ashley A Brenton, Lattha Souvannaseng, Kong Cheung, Michael Anishchenko, Aaron C Brault, Shirley Luckhart

**Affiliations:** 1 Department of Medical Microbiology and Immunology, School of Medicine, University of California Davis, 95616 Davis, CA, USA; 2 Division of Vector-Borne Diseases, National Center for Emerging Zoonotic Infectious Diseases, Centers for Disease Control and Prevention, 3156 Rampart Rd, 80521 Fort Collins, CO, USA

**Keywords:** Anopheles, Mosquito, MAPK, Plasmodium, Malaria, Single nucleotide polymorphism, Immunity

## Abstract

**Background:**

Susceptibility to *Plasmodium* infection in *Anopheles gambiae* has been proposed to result from naturally occurring polymorphisms that alter the strength of endogenous innate defenses. Despite the fact that some of these mutations are known to introduce non-synonymous substitutions in coding sequences, these mutations have largely been used to rationalize knockdown of associated target proteins to query the effects on parasite development in the mosquito host. Here, we assay the effects of engineered mutations on an immune signaling protein target that is known to control parasite sporogonic development. By this proof-of-principle work, we have established that naturally occurring mutations can be queried for their effects on mosquito protein function and on parasite development and that this important signaling pathway can be genetically manipulated to enhance mosquito resistance.

**Methods:**

We introduced SNPs into the *A. gambiae* MAPK kinase *MEK* to alter key residues in the N-terminal docking site (D-site), thus interfering with its ability to interact with the downstream kinase target ERK. ERK phosphorylation levels *in vitro* and *in vivo* were evaluated to confirm the effects of MEK D-site mutations. In addition, overexpression of various MEK D-site alleles was used to assess *P. berghei* infection in *A. gambiae.*

**Results:**

The MEK D-site contains conserved lysine residues predicted to mediate protein-protein interaction with ERK. As anticipated, each of the D-site mutations (K3M, K6M) suppressed ERK phosphorylation and this inhibition was significant when both mutations were present. Tissue-targeted overexpression of alleles encoding MEK D-site polymorphisms resulted in reduced ERK phosphorylation in the midgut of *A. gambiae*. Furthermore, as expected, inhibition of MEK-ERK signaling due to D-site mutations resulted in reduction in *P. berghei* development relative to infection in the presence of overexpressed catalytically active MEK.

**Conclusion:**

MEK-ERK signaling in *A. gambiae*, as in model organisms and humans, depends on the integrity of conserved key residues within the MEK D-site. Disruption of signal transmission via engineered SNPs provides a purposeful proof-of-principle model for the study of naturally occurring mutations that may be associated with mosquito resistance to parasite infection as well as an alternative genetic basis for manipulation of this important immune signaling pathway.

## Background

Malaria is caused by protozoan parasites of the genus *Plasmodium*, transmitted by female anopheline mosquitoes to humans during blood feeding. Prevention and treatment of the disease requires extensive efforts and coordination among the general public, health care organizations, and government agencies. Despite increased global efforts, malaria remains a top-ranked vector-borne disease and major global health concern, affecting over half of the world’s population every year [1]. Reports from populations in Sub-Saharan Africa recorded the highest numbers of cases and deaths estimated at 80% and 90% of the global burden, respectively, in 2012 [1]. In this region, *P. falciparum* is responsible for the largest number of infections and is the most deadly species, transmitted by *Anopheles gambiae*, the main mosquito vector.

A network of highly conserved cell signaling pathways controls malaria parasite development in and transmission by the anopheline mosquito host. Among these are the mitogen-activated protein kinase (MAPK) pathways, which function in growth, differentiation, and immune processes from nematodes to humans [2-5]. MAPKs function in multi-tiered sequential signaling cascades, in which an activated MAP4K phosphorylates and activates a MAP3K which, in turn, activates a downstream MAP2K, which activates a MAPK that can phosphorylate effector proteins or transcription factors to positively or negatively regulate a wide variety of cellular functions [6-8]. The subgroup involved in cellular proliferation and differentiation includes the extracellular signal-related kinase (ERK) and its upstream dual specificity MAPK/ERK kinase (MEK) [8].

Efficient propagation of MEK-ERK signaling requires a stable docking interaction between the upstream activating kinase and its downstream target. The N-terminal ERK docking site or D-site of MEK interfaces with the common docking or CD domain of ERK [9-13]. In humans, the first 32 or 36 residues of MEK1 or MEK2, respectively, comprise the D-site that mediates interaction with the common docking or CD domain of ERK [12,13]. The MEK D-site shares a conserved motif found in other MAPK-interacting proteins that includes a basic region, a ØA-X-ØB motif where Ø is leucine, isoleucine, or valine, and a hydrophobic-X-hydrophobic spacer region [14-17]. Deletion and mutational studies have revealed that the D-site is essential for enhancing the rate of MEK phosphorylation of ERK, and that the loss of the domain or substitution of the conserved basic and hydrophobic residues diminished the ability of MEK to bind to ERK [12,18]. In addition to the role of the MEK D-site in facilitating efficient activation, it is thought to tether ERK in the cytosol in resting cells [9].

The MEK-ERK signaling module plays a central role in the regulation of malaria parasite development in *Anopheles stephensi*, the Indian malaria mosquito [19-22]. In particular, human transforming growth factor-beta1 (TGF-β1) ingested with a *P. falciparum*-infected blood meal induces ERK activation in the midgut [20-22]. The provision of small molecule inhibitors of MEK in the blood meal reproducibly reduced ERK activation in the *A. stephensi* midgut and enhanced *nitric oxide synthase* (*NOS*) transcription within 24 h after infection, resulting in the production of inflammatory levels of reactive oxygen and nitrogen species in the midgut lumen [23] that are directly toxic to *P. falciparum*[24] and leading to significant reductions in oocyst numbers on the midgut epithelium [21]. Confirmation that small molecule inhibition of MEK can significantly reduce mosquito infectivity suggests that overexpression of altered MEK alleles could form the basis of a genetic strategy to generate parasite-resistant mosquitoes. Accordingly, we hypothesized that the introduction of non-synonymous single nucleotide polymorphisms (SNPs) into the highly conserved D-site of MEK could reduce ERK phosphorylation and decrease malaria parasite development in the mosquito host *in vivo*. Herein, we demonstrate that overexpression of a catalytically active MEK allele in *A. gambiae* cells *in vitro* resulted in enhanced ERK phosphorylation in these cells, while overexpression of a MEK allele with D-site mutations reduced ERK phosphorylation. Using a transient transformation strategy, midgut-specific overexpression of the same mutated MEK allele *in vivo* reduced ERK phosphorylation in this tissue and reduced development of naturally acquired *Plasmodium berghei in vivo*, suggesting for the first time that tissue-specific overexpression of mutated MEK could be used as the basis for a malaria transmission blocking strategy.

## Methods

### Cell culture, mosquito rearing and mosquito feeding

The immortalized *A. gambiae* Sua5B cell line [25] was maintained in Schneider’s medium (Invitrogen) with 10% heat-inactivated fetal bovine serum at 28°C. *Anopheles gambiae* (G3 strain) mosquitoes were reared and maintained at 27°C and 75% humidity. Mosquitoes were maintained under a 12 h light/dark cycle. Mosquito eggs were placed in water and fed 0.2% baker’s yeast on the day collected. After hatching, larvae were fed a mixture of liquid food containing 2% w/v powdered fish food (Sera Micron) and baker’s yeast in a 2:1 ratio, and Game Fish Chow pellet food (Purina). Adult mosquitoes were maintained on 10% sucrose solution-soaked cotton pads. All mosquito-rearing protocols were approved and in accord with regulatory guidelines and standards set by the Institutional Animal Care and Use Committee of the University of California, Davis. For *in vivo* studies, 3–5 d old female mosquitoes were allowed to feed for 30 min on artificial blood meals of washed human erythrocytes and heat-inactivated human serum provided through a Hemotek Insect Feeding System (Discovery Workshops).

### *MEK* allele plasmid construction and transfection for *in vitro* studies

**Figure 1:**
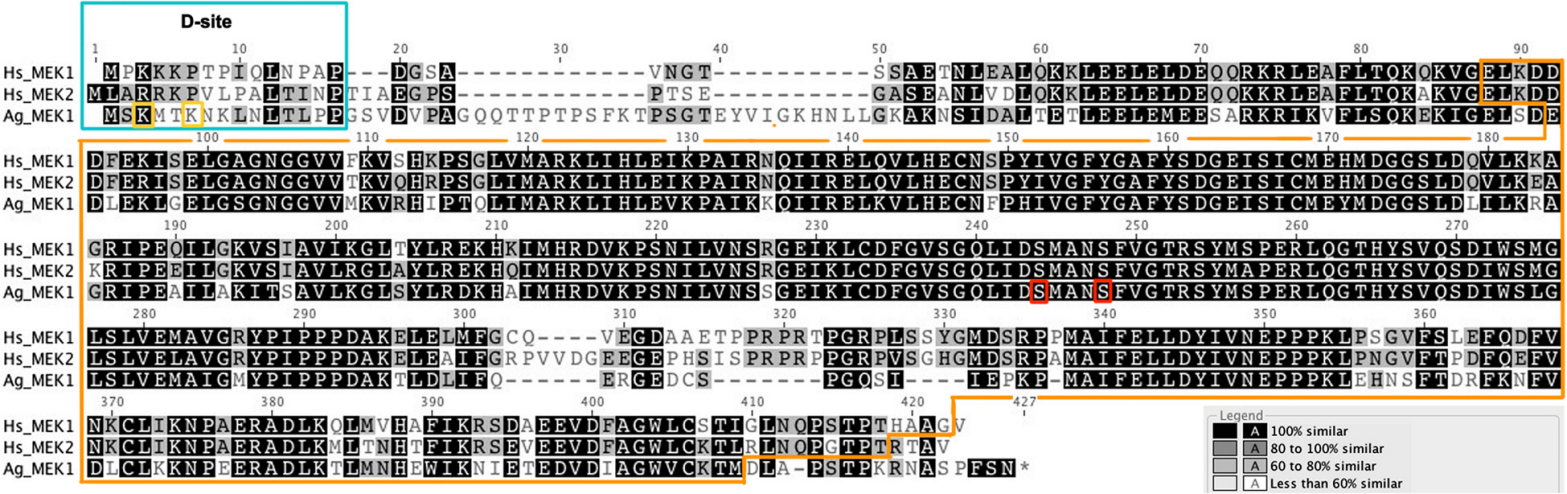
**Amino acid alignment of human MEK1 and MEK2 with *Anopheles gambiae *MEK.** Human (Hs) MEK1 and MEK2 and *A. gambiae* (Ag) MEK show significant overall conservation with high amino acid identity and similarity, including conservation in the docking site or D-site (blue box) and the catalytic domain (orange box). Lysine residues at positions 3 and 6 (yellow boxes) in the *A. gambiae* MEK allele were mutated to methionine (M). The key serine residues within the catalytic domain at positions 243 and 247 (red boxes) were mutated to aspartic acid (D) and glutamic acid (E), respectively. Human MEK1 [Genbank: NP_002746], human MEK2 [Genbank: NP_109587] and *A. gambiae* MEK1 [Genbank: XP_322064] protein sequences were aligned using the MUSCLE method with default settings in Geneious [26].

The complete mRNA sequence of *A. gambiae MEK* [GenBank: XM_322064] in the pDREAM 2.1 vector (Genscript) (wild type MEK or wtMEK) was used to generate five additional plasmids encoding *MEK* mRNA with various combinations of SNPs: pMEK1, pMEK2, pMEK3, pMEK4 and pMEK5 (see Table 1 for a summary of these mutations). In brief, SNPs were introduced at codon positions 3 and 6 to convert lysines (K) to methionines (M) and at positions 243 and 247 to convert serines (S) to glutamic acid (E) and aspartic acid (D), respectively (Figure 1).

**Table 1:** pMEK plasmid nucleotide changes to D-site lysines and catalytic site serines

**Domain**	**Codon Position**	**wtMEK**	**pMEK1**	**pMEK2**	**pMEK3**	**pMEK4**	**pMEK5**
**Docking (D)-Site**	**3**	AAA (K)	AAA (K)	AAA (K)	ATG (M)*	AAA (K)	ATG (M)*
	**6**	AAA (K)	AAA (K)	AAA (K)	AAA (K)	ATG (M)*	ATG (M)*
**Catalytic**	**243**	TCA (S)	GAA (E)*	GAA (E)*	GAA (E)*	GAA (E)*	GAA (E)*
	**247**	TCT (S)	TCT (S)	GAT (D)*	GAT (D)*	GAT (D)*	GAT (D)*

Mutations at positions 3 and 6 in the D-site were introduced to disrupt MEK-ERK interaction and corresponding phosphorylation of ERK. Mutations at positions 243 and 247 mimic MEK phosphorylation and, hence, activation. The codons are shown with encoded amino acids in parentheses. wtMEK = wild type MEK; amino acid substitutions are noted with asterisks.

To introduce SNPs into the *MEK*-encoding sequence, paired synthetic primers that encoded the desired mutations were synthesized (See Table 2 for primer sequences; Sigma-Aldrich) and utilized for mutagenic primer-directed replication of both plasmid strands with high-fidelity *PfuUltra* DNA polymerase (Agilent). The following conditions were used for plasmid replication: 15–17 cycles of denaturation at 95°C for 30 sec, primer annealing for 1 min at 55°C, followed by extension at 68°C for 1 min per 1 kb amplified. The products were treated with endonuclease DpnI (New England BioLabs) for digestion of the parental DNA template and purification of the selected mutation-encoding synthesized DNA. The nicked synthesized plasmid DNAs with the desired mutations were transformed into *E. coli* TOP10 chemically competent cells (Invitrogen). Eight to ten transformed colonies for every desired mutation were screened for plasmid DNA using the Qiagen Miniprep Kit and the manufacturer’s instructions (Qiagen). Among those, four to five plasmids were sequenced for confirmation of the introduced functional nucleotide changes (CDC Sequencing Facility, Fort Collins, CO).

**Table 2:** Primer sequences for site-directed mutagenesis

**Plasmid constructs**	**Resulting amino acid mutations**	**Primer sequence 5′ ➔ 3′**
*Docking domain mutations*		
pMEK3	K3M	F: GACGACGACAAGATGAGTA**TG**ATGACAAAAAACAAACTTAA
		R: TTAAGTTTGTTTTTTGTCAT**CA**TACTCATCTTGTCGTCGTC
pMEK4	K6M	F: CAAGATGAGTAAAATGACAA**TG**AACAAACTTAATTTGACGTTG
		R: CAACGTCAAATTAAGTTTGTT**CA**TTGTCATTTTACTCATCTTG
pMEK5	K3M	F: GACGACGACAAGATGAGTA**TG**ATGACAA**TG**AACAAACTTAATTTGACGTTG
	K6M	R: CAACGTCAAATTAAGTTTGTT**CA**TTGTCAT**CA**TACTCATCTTGTCGTCGTC
*Catalytic domain mutations*		
pMEK1	S243E	F: GATTGAT**GA**AATGGCCAATTCTTTTGTAGGTACTCGAAG
		R: CTTCGAGTACCTACAAAAGAATTGGCCATT**TC**ATCAATC
pMEK2	S243E	F: GATTTCGGCGTTTCCGGTCAGTTGATTGAT**GA**AATGGCCAAT**GA**TTTTGTAGGTACTCGAAG
pMEK3		
pMEK4	S247D	R: CTTCGAGTACCTACAAAA**TC**ATTGGCCATT**TC**ATCAATCAACTGACCGGAAACGCCGAAATC
pMEK5		

Paired synthetic primers encoding the engineered SNPs (bold) were used to introduce mutations into the *MEK*-encoding sequence through mutagenic primer-directed replication of both plasmid strands.

*MEK*-encoding plasmids were transfected into *A. gambiae* Sua5B cells using Effectene Reagent (Qiagen) and the manufacturer’s recommended protocol. In brief, 1×10^6^ Sua5B cells in 2 mL medium were plated in 6-well tissue culture plates overnight at 28°C. At 24 h after plating, cells were transfected with 0.6 μg of plasmid DNA and incubated at 28°C. At 36 h post-transfection, medium was removed and cells were washed with ice-cold phosphate buffered saline (PBS) in preparation for immunoblotting.

### *MEK* allele plasmid construction for *in vivo* studies and microinjection of female *A. gambiae*

The plasmid for transgene overexpression in adult female *A. gambiae* was described previously [27]. To ensure midgut-specific expression of the transgene post-blood feeding, the *A. gambiae* carboxypeptidase promoter was engineered into the plasmid [28,29]. The MEK inserts wtMEK, pMEK2 and pMEK5 were cloned into the plasmid using 5′-PstI and 3′-SalI restriction sites.

For each experiment, at least twenty laboratory reared 3–5 d old female *A. gambiae* were allowed to feed for 30 min on artificial blood meals at 16–24 h prior to *MEK*-encoding plasmid inoculation. For our studies, a mixture of 0.5 μg/μl *MEK*-encoding plasmid DNA, the *in vivo* transfection reagent jetPEI^TM^ (Polyplus-transfection Inc.) and glucose at a final concentration of 5% [27] was injected into the hemocoel of vitellogenic females (0.1 to 0.5 μg DNA/female) using the Nanoject II Auto Nanoliter Injector (Drummond Scientific Company). At 24 h post injection, the mosquitoes were provided small cups of water for oviposition. F0 eggs were collected and reared through to the adult stage.

### Mosquito cell and tissue preparation and immunoblotting

To harvest proteins from *A. gambiae* Sua5B cells, cells were lysed in 200 μl cell lysis buffer (10 mM Tris–HCl pH 7.4, 1 mM EDTA, 100 mM NaCl, 1 mM NaF, 1 mM EGTA, 2 mM Na_3_VO_4_ 20 mM Na_4_P_2_O_7_, 0.1% SDS, 1% Triton X-100, 0.5% sodium deoxycholate, 1 mM phenylmethylsulfonyl fluoride, 10% glycerol, 60 mg/mL aprotinin, 10 mg/ml leupeptin, 1 mg/ml pepstatin, 1 mg/ml calyculin A). Cellular debris was removed by centrifugation at 14,000 × *g* for 10 min at 4°C. The resulting supernatants were mixed with Laemmli sample buffer (125 mM Tris–HCl pH 6.8, 10% glycerol, 10% SDS, 0.006% bromophenol blue, 130 mM dithiothreitol) and the proteins were denatured at 95°C for 4 min prior to electrophoresis.

Mosquito midguts were dissected into PBS and mixed to release blood, if any, by pipetting up and down. The midguts were washed in a filter column fitted with a fine mesh with a mixture of protease and phosphatase inhibitor cocktails (Sigma) in ice-cold PBS until all of the blood was removed. Fresh PBS mixture was added to loosen the midgut tissue from the filter, transferred to a fresh tube, and then centrifuged and prepared for electrophoresis as for cell culture lysate above.

Proteins were separated on 10% SDS-PAGE polyacrylamide gels at 135 V for 1 h, 50 min. Proteins were transferred to nitrocellulose membranes (Bio-Rad Laboratories) for 1 h, 15 min at 7 V. Coomassie blue staining of the polyacrylamide gel was used to visually assess consistency of protein loading. Membranes were blocked in nonfat dry milk (5% w/v) in 1X Tris-buffered saline (TBS; pH 7.0) containing 0.1% Tween (TBS-T) for 1 h at room temperature, and then reacted overnight in primary antibody at 4°C. The membrane was washed 3 times, 5 min each with 1X TBS-T followed by incubation with appropriate secondary antibody 4°C overnight. The membrane was washed again 3 times, 5 min each with 1X TBS-T and then incubated in SuperSignal West Dura Extended Duration Substrate (Pierce). The Kodak Image Station 4000MM Pro Imaging System (Carestream Health, Inc.) was used to capture the image of the membrane and Quantity One (Bio-Rad Laboratories) software was used for densitometry analysis of the antibody-bound proteins. Levels of phosphorylated ERK (pERK) in Sua5B cells for each treatment were normalized to total ERK levels for protein loading and then normalized to pERK levels in the control cells transfected with wtMEK plasmid construct for *in vitro* experiments. Levels of pERK in the midgut for each group of *A. gambiae* were normalized to GAPDH levels and then to pERK levels in control mosquitoes transformed with wtMEK plasmid construct for *in vivo* experiments.

Primary antibodies and dilutions included anti-FLAG-M2 (A2220; Sigma-Aldrich) (1:7,500), anti-GAPDH (G9545; Sigma-Aldrich) (1:10,000), anti-diphosphorylated ERK (pERK) (M8159; Sigma-Aldrich) (1:10,000) and anti-ERK1/2 (total ERK) (9102; Cell Signaling Technology) (1:1,250). Anti-rabbit IgG-peroxidase (A0545; Sigma-Aldrich) (pERK 1:20,000; FLAG 1:2,000) and anti-mouse IgG-peroxidase (A9044; Sigma-Aldrich) (GAPDH and total ERK 1:20,000) were used as secondary antibodies for immunoblotting.

### Real-time quantitative PCR

Total RNA was isolated from dissected individual midguts and carcasses (all tissue remaining after dissection) using TRIzol reagent (Invitrogen) and genomic DNA was removed using TURBO DNA-*free* (Invitrogen). Quantitative RT-PCR was performed on an ABI Prism 7300 Sequence Detection System (Applied Biosystems). Primers and Taqman probes (Applied Biosystems) were designed to distinguish over-expressed alleles from endogenous *A. gambiae* MEK mRNA: *MEK*-RT forward, 5′CCGAGCAACATTCTTGTAAATAGCAGTGG3′; *MEK*-RT reverse, 5′AAGCGCTCGGGCGACATATAAC3′; *S7* forward, 3′GAAGGCCTTCCAGAAGGTACAGA3′; *S7* reverse 5′CATCGGTTTGGGCAGAATG3′; *wtMEK* probe, 6FAM-GATTCAATGGCCAATTCTTTTGTAGG-MGBNFQ; *MEK2/5* probe, 6FAM-GATGAAATGGCCAATGATTTTGTAGG-MGBNFQ; and *S7* probe, VIC-AGAAGTTCTCCGGCAAGCACGTCGT-6-carboxytetramethylrhodamine. Amplification conditions were defined as reverse transcription at 48°C for 30 min, AmpliTaq Gold activation at 95°C for 10 min, and then 40 cycles of denaturation at 95°C for 15 sec and annealing/extension at 60°C for 1 min.

### Laboratory infection of mice with *P. berghei* and mosquito blood feeding

Female CD1 mice were infected with *P. berghei* for transmission to *A. gambiae*. When parasitemia reached 5-10% of peripheral red blood cells (typically at 4 d post-infection), mice were anesthetized and exposed to mosquitoes for feeding. Thirty 3–5 d old F0 female mosquitoes transformed for midgut-specific overexpression of wtMEK, pMEK2 or pMEK5 were aspirated into individual cartons. Non-transformed *A. gambiae* females in a fourth carton served as an additional control. Mosquitoes were allowed to rest for 24 h and starved 2–4 h prior to blood feeding on anesthetized *P. berghei-*infected mice for 30 min. All non-blood fed females were removed from the containers using a mechanical aspirator while the remainder were maintained at 19°C and 80% humidity. At 12 d post-blood feeding, mosquito midguts were dissected in PBS and stained with mercurochrome for direct counting of *P. berghei* oocysts.

Protocols involving the culture and handling of *P. berghei* were approved and in accord with regulatory guidelines and standards set by the Biological Safety Administrative Advisory Committee of the University of California, Davis. Experiments involving the use of animals were reviewed and deemed to be in accord with all relevant institutional policies and federal guidelines by the UC Davis Institutional Animal Care and Use Committee (protocol #17619, expiration 26 June 2014).

### Statistical analyses

Differences in levels of ERK phosphorylation in Sua5B cells *in vitro* were analyzed by ANOVA (α = 0.05) for overall significance and by Bonferroni’s test for pairwise comparisons of means. Differences in exogenous *MEK* allele expression *in vivo* were analyzed by ANOVA (α = 0.05) and by Bonferroni’s test for pairwise comparisons of means. A Pearson’s r test was performed to assess the relationship between docking site mutations and ERK phosphorylation levels in midgut tissue of transformed F0 females (α = 0.05). Significant differences in control (non-transformed mosquitoes) mean oocyst counts among replicates were determined by one-way ANOVA (α = 0.05); no differences were detected, so replicates were combined for analyses. Significant differences in oocyst counts from combined replicates were detected by unpaired t-tests (α = 0.05). All calculations were performed using GraphPad Prism version 5.02 for Windows (GraphPad Software, San Diego, California USA).

## Results

### S243E and S247D mutations in the catalytic core of *A. gambiae* MEK mimicked kinase activation in Sua5B cells *in vitro*

**Figure 2:**
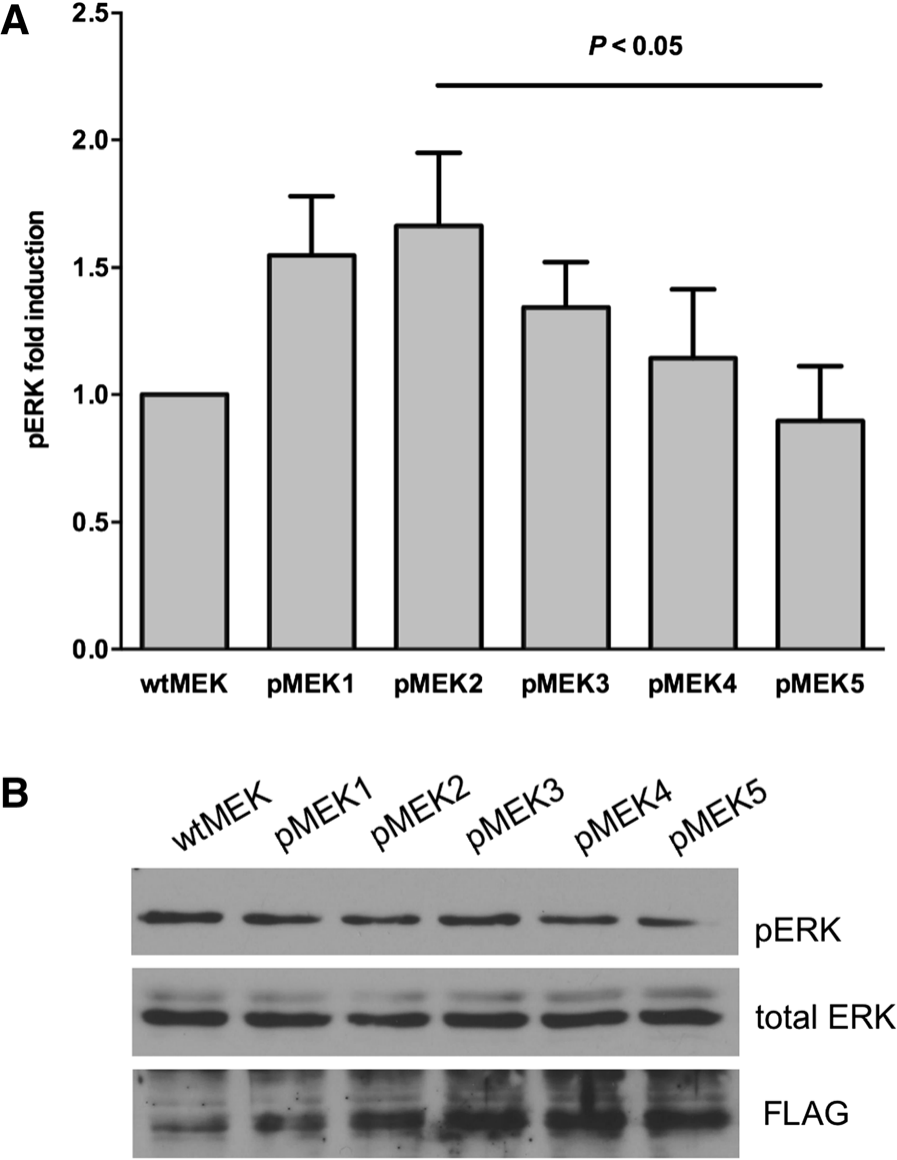
**SNPs within MEK D-site and catalytic domain alter *Anopheles gambiae *ERK phosphorylation *in vitro*. A**, ERK phosphorylation (pERK) levels in Sua5B cells transfected with pMEK1-5 were first normalized to total ERK, and then to pERK levels in control cells transfected with the unaltered allele (wtMEK; set here as 1.0). The introduction of S243E and S243E/S247D mutations in pMEK1 and pMEK2, respectively, increased pERK levels, although not significantly, in transfected cells relative to control cells. However, pERK levels in cells transfected with catalytically active MEK K3M/K6M (pMEK5) were significantly lower than levels observed in cells transfected with catalytically active pMEK2 (ANOVA, Bonferroni’s test for pairwise comparisons, *P* < 0.05). Data are represented as means ± SEMs (N = 5). **B**, Representative western blots showing phosphorylated ERK, total ERK, and FLAG in Sua5B cells transfected with plasmids as in A. Detection of FLAG confirmed MEK overexpression.

The substitution mutations in *A. gambiae* MEK (S243E and S247D; Figure 1, Table 1) resulted in negatively charged residues that mimic phosphorylation in the absence of exogenous stimuli and, therefore, mimicked kinase activation as described for the analogous mammalian MEK mutations S218E/S222D [30,31]. Specifically, *A. gambiae* Sua5B cells that were transfected with pMEK1 (S243E) or with pMEK2 (S243E/S247D) had 50-70% higher levels of ERK phosphorylation relative to cells transfected with wtMEK plasmid, which encoded the unaltered *MEK* allele (Figure 2A). ERK phosphorylation levels were approximately 20% higher, albeit not significantly, in cells transfected with pMEK2 relative to cells transfected with pMEK1 (Figure 2A), suggesting a modest additive effect of the activating mutations [30,31]. These increases in ERK phosphorylation were comparable to human TGF-β1-induced ERK phosphorylation in *A. gambiae* cells (70% to 2.5-fold above control) [3], but were substantially lower than those observed following analogous overexpression studies in mammalian cells. In particular, overexpression of human MEK S218E/S222D in human kidney 293 or monkey kidney COS-7 cells increased ERK activity by more than 100-fold above wild type MEK levels [30,31]. However, background ERK phosphorylation in the absence of stimulation in both 293 and COS-7 cells is nearly undetectable [30,31], whereas previously observed basal ERK phosphorylation levels in *A. gambiae* cells in the absence of treatment [3] are nearly comparable to levels following transfection with wtMEK (Figure 2B).

### D-site mutations in catalytically active *A. gambiae* MEK reduced ERK phosphorylation in Sua5B cells *in vitro*

**Figure 3:**
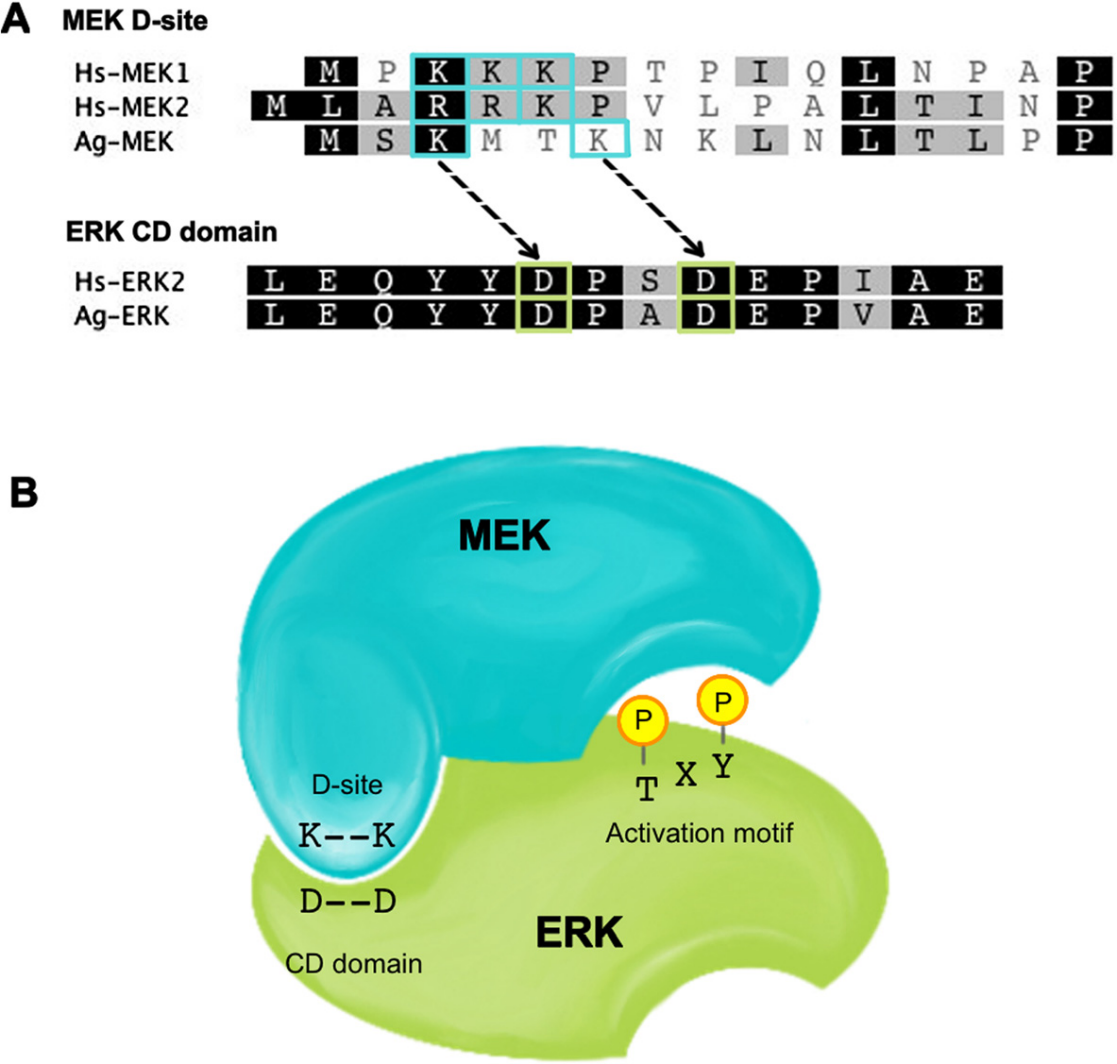
**MEK-ERK signaling transmission is dependent on a docking interaction between key residues. A**, The *A. gambiae* MEK (Ag-MEK) D-site shares similarities with human MEK D-sites (Hs-MEK1 and Hs-MEK2). The key D-site residues (blue boxes) are predicted to interact with aspartic acid residues within the CD-domain of ERK (green boxes). *A. gambiae* ERK [Genbank: XP_319983] and human ERK2 [Genbank: NP_002736] protein sequences were aligned using the MUSCLE method with default settings in Geneious [26]. **B**, A schematic illustration of MEK-ERK protein interaction. The key lysine residues within the MEK D-site interact with the aspartic acids within the CD-domain of ERK. Following the binding of the two proteins, MEK phosphorylates threonine and tyrosine within the activation motif of ERK.

Based on functional interactions of MEK and ERK in mammalian cells [16], we predicted that conserved lysine residues K3 and K6 encoded in the *A. gambiae* MEK D-site should interact directly with two conserved aspartic acids in the CD domain of ERK (Figure 3). As such, MEK S243E/S247D is catalytically active, but the addition of K3M and K6M mutations would be expected to block the interaction of activated MEK with ERK and, hence, block ERK activation (Table 1). While not significant, overexpression of catalytically active MEK K3M (pMEK3) or catalytically active MEK K6M (pMEK4) in *A. gambiae* Sua5B cells reduced ERK phosphorylation relative to cells overexpressing catalytically active MEK (pMEK2; Figure 2A). Furthermore, overexpression of catalytically active MEK K3M/K6M (pMEK5) resulted in a significant reduction in ERK phosphorylation relative to cells that were transfected with pMEK2 (Figure 2A), suggesting that D-site lysine residues in *A. gambiae* are essential for functional docking and phosphorylation of ERK by MEK.

### Transovarially acquired pMEK2 and pMEK5 resulted in midgut-biased transgene overexpression in F0 mosquitoes

Consumption of blood initiate vitellogenesis in the female mosquito during which the fat body produces yolk protein precursors that are absorbed by the developing oocytes [32,33]. Peng *et al*. [27] exploited this physiology to develop a “vertical DNA vector delivery method” to transiently manipulate gene expression in F0 offspring of plasmid-injected female mosquitoes. We used this strategy to investigate whether MEK D-site mutations could alter ERK phosphorylation in the *A. gambiae* midgut. Specifically, pMEK2 and pMEK5 plasmids were microinjected into the hemocoels of vitellogenic female mosquitoes that had fed on blood 16–24 h earlier. Following injection, mosquitoes were allowed to oviposit and eggs (F0) were collected for rearing.

**Figure 4:**
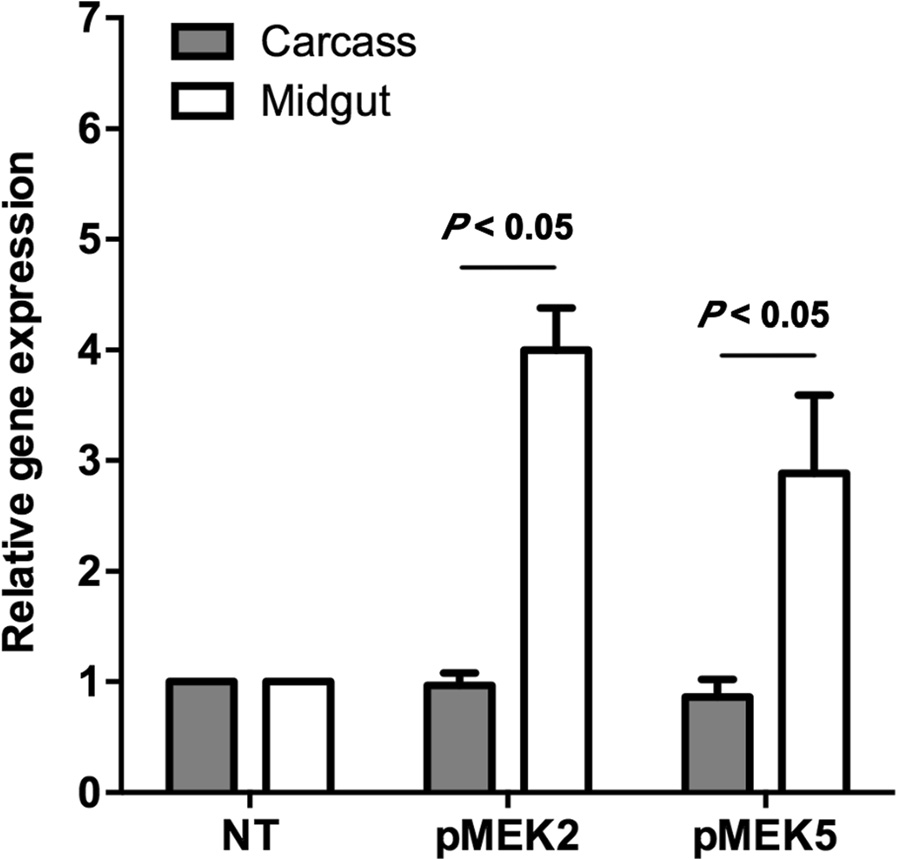
**Midgut-specific overexpression of exogenous variant *MEK *alleles was confirmed by qRT-PCR.** Total RNA was isolated from the midguts or carcasses of non-transformed (NT) female mosquitoes and female mosquitoes transformed with either pMEK2 or pMEK5 for qRT-PCR with probes that distinguished between exogenous variant and endogenous *MEK* alleles. Relative to levels of endogenous *MEK* mRNA in NT females, expression levels of exogenous variant *MEK* allele were four- or three-fold higher (*P* < 0.05) in midguts of females transformed with plasmids encoding catalytically active MEK (pMEK2) or catalytically active MEK K3M/K6M (pMEK5), respectively. Variant *MEK* allele expression levels in the carcass of transformed mosquitoes were comparable to endogenous *MEK* allele expression levels in NT female mosquitoes. Five independent mosquito cohorts were used in this experiment.

To confirm midgut-specific overexpression of the variant *MEK* alleles, 4 d old F0 adult female offspring of plasmid-injected *A. gambiae* were allowed to feed on blood for 30 min. Age-matched F0 offspring from mosquitoes from the same cohorts that were not injected with plasmid and, hence, not transformed were fed alongside transformed F0 females as controls to assess specificity of transcript detection. At 2 h post-feeding, midguts from six female *A. gambiae* in each group were dissected into PBS. TaqMan qRT-PCR with probes specific to exogenous variant *MEK* alleles revealed no detectable signals from midguts or carcasses of non-transformed F0 females; hence, relative levels of endogenous *MEK* mRNA in non-transformed females (NT) were used for comparison to variant *MEK* allele expression in transformed F0 females (pMEK2, pMEK5; Figure 4). From replicated F0 cohorts, midgut expression of mRNAs encoding catalytically active MEK (pMEK2) and catalytically active MEK K3M/K6M (pMEK5) were detected at levels four- and three-fold higher (*P* < 0.05) than endogenous *MEK* mRNA levels in non-transformed mosquitoes (Figure 4). Surprisingly, expression of exogenous variant *MEK* alleles in the carcass appeared to be equivalent to endogenous *MEK* mRNA levels in the same tissue in non-transformed F0 females (compare carcass levels of *pMEK2* and *pMEK5* to carcass levels of endogenous *MEK* in non-transformed females; Figure 4). While variant *MEK* allele probes specifically detected transcripts in transformed mosquitoes, we cannot exclude the possibility that some level of endogenous *MEK* mRNA is also detected by these probes, which would explain signal detection in the carcass of transformed F0 females despite use of a midgut-specific promoter [29,34].

### Midgut-directed overexpression of *MEK* alleles with D-site polymorphisms decreased ERK phosphorylation

**Figure 5:**
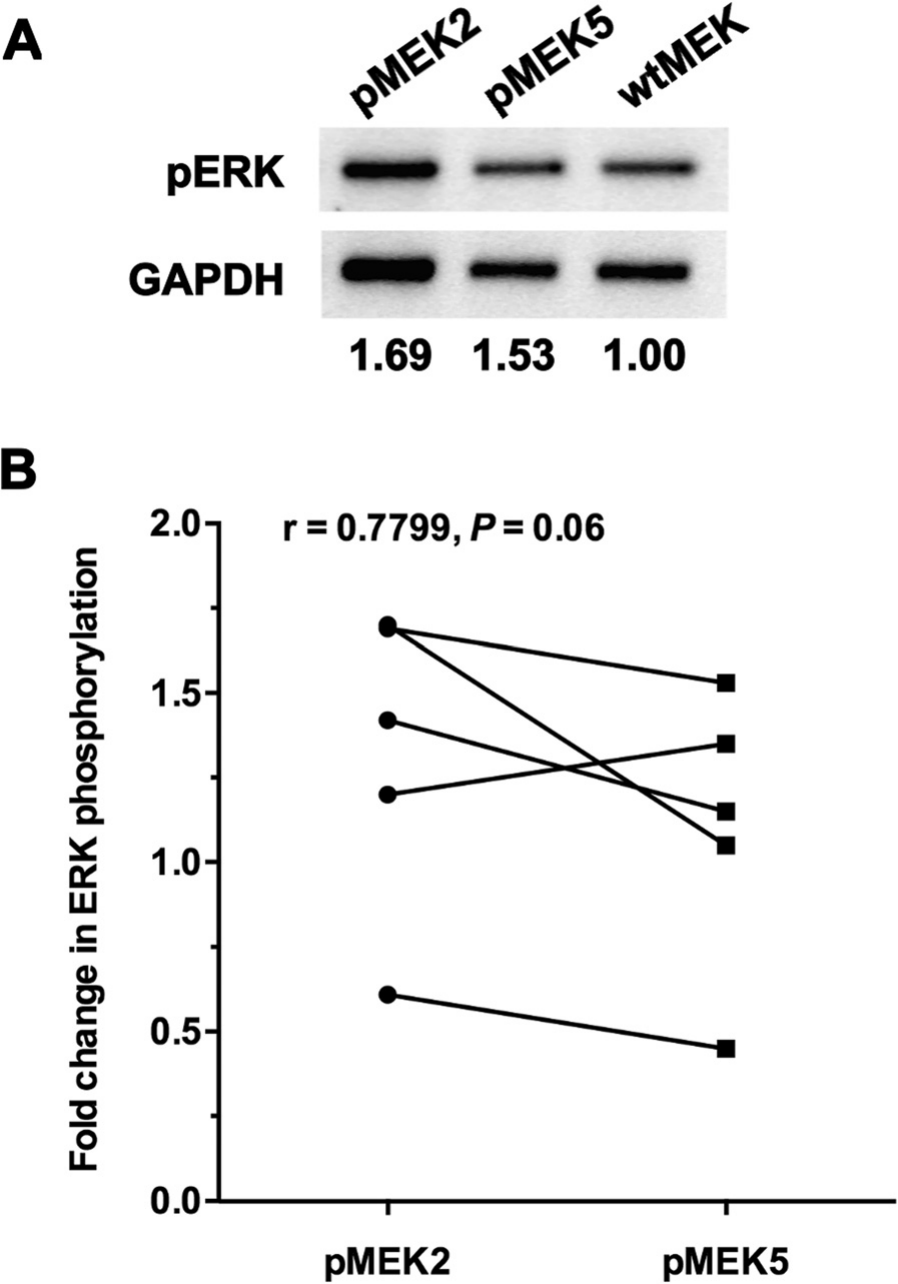
**D-site mutations were correlated with repressed midgut ERK phosphorylation levels in *A. gambiae *compared to mosquitoes transformed with catalytically active MEK. A**, Representative western blot of ERK phosphorylation in midgut epithelia from transformed mosquitoes examined at 2 h after blood feeding. Values below the blots indicate relative fold change in pERK levels in the midgut for each group of *A. gambiae*. Phosphorylated ERK levels were normalized to GAPDH and then to pERK levels in wtMEK, which is set at 1.00. **B**, Fold changes in ERK phosphorylation levels are indicated as trendlines between samples from matched treatment groups for pMEK2 and pMEK5 overexpression. Note that in one replicate, relative values of phosphorylation for pMEK2 and pMEK5 transformed *A. stephensi* were below the level of controls (e.g., < 1), but nonetheless revealed the same trend of decreasing phosphorylation. A Pearson’s r test was performed to assess the relationship between docking site mutations and ERK phosphorylation levels in midgut tissue of transformed F0 females (r = 0.7799, *P* = 0.06).

Based on midgut-biased variant *MEK* mRNA expression in transformed F0 mosquitoes (Figure 4), we examined this tissue from transformed F0 females for relative levels of ERK phosphorylation. At 2 h post-blood meal, 30–45 midguts from each group were dissected, pooled and processed for immunoblotting (Figure 5A). As shown in Figure 5B, the response to pMEK2 transformation was correlated with the response to pMEK5 across replicates (r = 0.7799). In particular, 4 of 5 replicates showed a reduction in ERK phosphorylation levels in midgut epithelia of mosquitoes that overexpressed the catalytically active but docking deficient MEK (pMEK5) relative to levels in mosquitoes that overexpressed catalytically active MEK (pMEK2; *P* = 0.06). We reasoned that a mean reduction of approximately 23% in phosphorylated ERK levels for 4 of 5 replicates, although marginally not significant, could be biologically significant given our previous observations that incomplete inhibition of TGF-β1-induced midgut ERK phosphorylation by the MEK inhibitor PD98059 [21] could result in significant inhibition of *P. falciparum* growth in *A. stephensi*.

### Midgut-directed overexpression of *MEK* alleles with D-site polymorphisms decreased *P. berghei* development in *A. gambiae*

**Figure 6:**
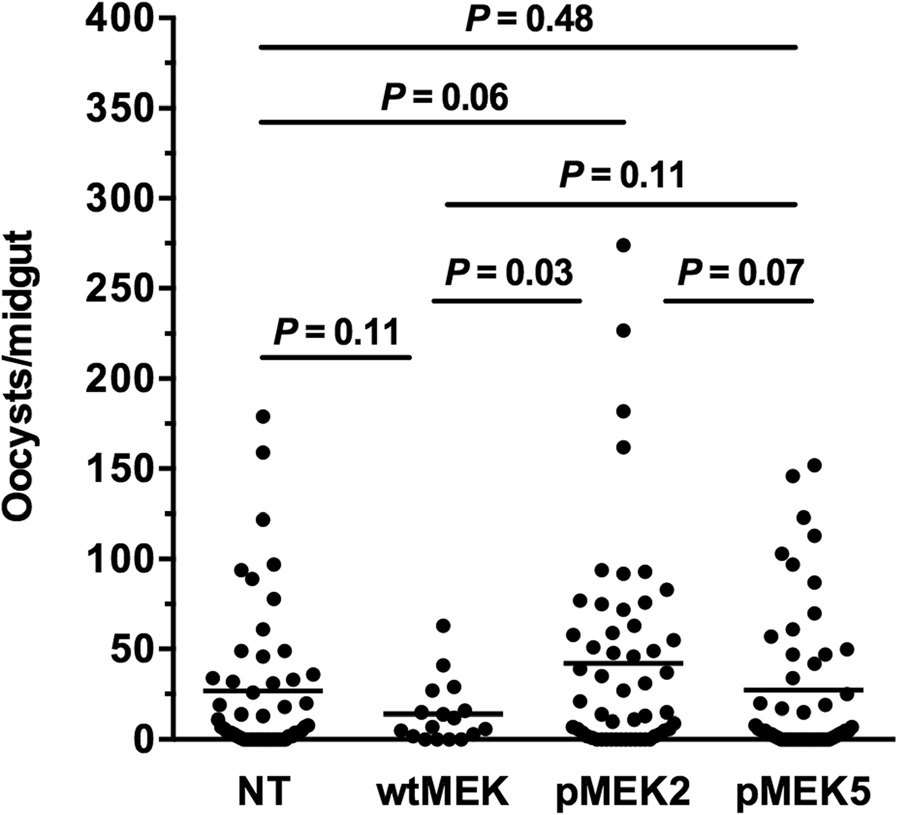
**Midgut-directed overexpression of *MEK *alleles with D-site polymorphisms decreased *P. berghei *oocyst numbers in *A. gambiae *females *in vivo.*** We allowed 3–5 d old female mosquitoes transformed to overexpress wtMEK, pMEK2 or pMEK5 to feed on *P. berghei*-infected mice. Twelve days after infection, mosquito midguts were dissected and *P. berghei* oocysts were counted. The experiment was replicated three times. There were no significant differences in NT mean oocyst counts among replicates (one-way ANOVA), so replicates were combined for analysis. Midgut-specific overexpression of catalytically active MEK (pMEK2) resulted in a significant increase in the number of oocysts per midgut compared to mosquitoes overexpressing wtMEK (*P* = 0.03). Mutation of the key residues in the D-site (pMEK5), however, resulted in oocyst development that was not significantly different from NT females (P = 0.48) and females transformed with wtMEK (*P* = 0.11).

To determine whether overexpression of catalytically active MEK with D-site mutations would result in an infection phenotype similar to that induced by PD98059 inhibition of MEK [21], we allowed 3–5 d old female mosquitoes transformed for midgut-specific overexpression of wtMEK, pMEK2 or pMEK5 to feed on *P. berghei*-infected mice for 30 min (Figure 6). As expected, mosquitoes overexpressing catalytically active MEK (pMEK2) developed a significantly greater number of oocysts per midgut (42.2 ± 8.0) than did mosquitoes overexpressing wtMEK (14.1 ± 1.2 oocysts; *P* = 0.03). Oocyst counts in mosquitoes overexpressing wtMEK were not significantly different from non-transformed (NT) mosquitoes (*P* = 0.11). The introduction of D-site mutations (pMEK5) reduced oocyst development (28.0 ± 5.8 oocysts) relative to mosquitoes transformed with pMEK2 (*P* = 0.07) to levels that were not different from mosquitoes overexpressing wtMEK (*P* = 0.11) and non-transformed mosquitoes (*P* = 0.48). These results confirm our previous observations that MEK-ERK signaling regulates parasite development and indicate that this regulation is dependent on MEK docking functionality.

## Discussion

In this study, we used a vertical DNA vector delivery method adapted from Peng *et al*. [27] to demonstrate that mutations in the D-site of MEK disrupt downstream ERK phosphorylation in *A. gambiae* cells *in vitro* and *in vivo*. Further, these mutations can alter the success of malaria parasite infection in transformed F0 mosquitoes. Specifically, K3M and K6M mutations in the D-site (pMEK5; Table 1) significantly repressed ERK phosphorylation relative to cells or tissue in which catalytically active MEK was overexpressed (pMEK2; Figures 2, 5). These data suggest that the MEK D-site has conserved functionality in an insect of public health importance.

Because of the critical involvement of MEK-ERK signaling in a number of chronic diseases and cancer, there has been significant interest in therapeutic approaches to disrupt the docking interaction of MEK and ERK, including the use of small molecule inhibitors [35], the introduction of mutations into the MEK D-site, and transfection of short blocking peptides that bind to the CD domain of ERK [36]. In mammals, disruption of the MEK D-site can also facilitate immune evasion by pathogens. For example, the anthrax lethal factor (ALF) of *Bacillus anthracis* impairs host cell immune activation during early infection through cleavage of the MEK D-site by anthrax lethal protease [37-39]. In the context of cancer, ALF-mediated cleavage of the D-site regulates both cell survival and growth. Specifically, ALF-mediated inhibition of MAPK signaling can trigger melanoma cell apoptosis and suppress the progression of renal cell carcinoma [40-42]. From these studies, we suggest that enhanced disruption of the D-site of MEK as a strategy to inhibit disease-associated MEK-ERK signaling could be applied to the development of transmission blocking strategies for malaria.

Our engineered mutations in the MEK D-site did not appear to exert a dominant negative effect, but induced an incomplete albeit functional block of ERK phosphorylation, much like the use of small molecule MEK inhibitors. Based on these observations, we suggest that mutated MEK did not wholly outcompete endogenous MEK to block signal transmission. Alternatively, inhibition may have been more effective than was apparent because of feedback regulation. In particular, the MEK inhibitor PD98059 can interfere with ERK-dependent negative feedback regulation. Specifically, the Grb2-SOS complex is recruited to activate membrane-bound Ras, but ERK phosphorylation of SOS causes the complex to dissociate [43]. Following treatment with PD98059, ERK-dependent SOS phosphorylation is blocked, resulting in prolonged Ras activation in insulin- and epidermal growth factor (EGF)-treated human insulin receptor expressing rat cells [44]. Thus, blocking the signaling interaction of MEK and ERK can impair feedback regulation to partially restore ERK phosphorylation.

In addition to canonical signaling through Ras-Raf-MEK, networked signaling pathways can provide alternative targets for manipulation of ERK phosphorylation. For example, treatment of human breast cancer T47D cells with the MEK inhibitor U0126 in combination with the phosphoinositide 3-kinase/Akt inhibitor wortmannin synergistically suppressed EGF-induced ERK activation relative to treatment with U0126 alone [45]. Similarly, overexpression of phosphatase and tensin homolog (PTEN), which functions as an endogenous inhibitor of Akt, reduced basal levels of midgut ERK phosphorylation relative to control *A. stephensi*, but had no effect on insulin-induced ERK activation relative to controls [34], suggesting that inhibition of Akt-dependent ERK signaling could be targeted to additively reduce ERK activation in the mosquito. The activation of endogenous ERK inhibitors has also proved successful in suppressing ERK activation with measurable biological effects. For example, overexpression of MAPK phosphatase MKP-3, which specifically targets ERK for dephosphorylation, induced hepatic gluconeogenesis and increased fasting blood glucose levels in lean mice, suggesting that MKP-3 could be targeted therapeutically for type 2 diabetes [46]. Similarly, *Drosophila melanogaster* MKP-3 is an endogenous regulator of ERK phosphorylation that is indispensable to fly embryonic development [47], indicating that genetic manipulation of MKP-3 can provide highly conserved control of important biological responses to ERK phosphorylation. Finally, the MKPs are an intense focus of development of therapeutic small molecule inhibitors and activators given the critical roles for MAPKs in various chronic and inflammatory human diseases [48], suggesting that analogous discovery and applications for enhancing MKP activity in mosquitoes are both possible and likely to be successful.

Naturally occurring SNPs within *A. gambiae* immune genes have been found to be associated with parasite infection, including *TOLL5B* and *ILP3*[49] as well as *Sp SNAKElike* and *TOLL6*[50]. Indeed, it has been proposed that a breakdown in mosquito innate immunity is responsible for susceptibility to parasite infection [51], raising the possibility of undertaking studies searching for naturally occurring mutations in immune signaling genes. Most recently, Li *et al.*[52] identified nonsynonymous SNPs in *A. gambiae adenosine deaminase* (*AgADA*)*, fibrinogen-related protein 1* (*FREP1*) and *fibrinogen-related protein 30* (*FBN30*). RNAi-mediated silencing of *FREP1* resulted in a significant decrease in infection prevalence while *FBN30* transcript ablation increased infection intensity two-fold relative to controls [52]. While these association studies support the role of natural genetic variation in the control of *Plasmodium* development, mutations such as these have thus far been used only to identify target genes for which to query the effects of RNAi-mediated silencing on infection phenotype. However, many immune gene products require protein-protein interaction to form complexes or post-translational modification to mediate specific cellular functions. As such, the work presented here indicates that functional SNP studies can be extended to determine the mechanisms by which coding sequence mutations specifically impact infection phenotype.

## Conclusion

We have established proof-of-principle for the functional analysis of SNPs on protein function and infection susceptibility in *A. gambiae*. In particular, we have demonstrated that engineered mutations in *A. gambiae* MEK recapitulate the effects of small molecule inhibition of MEK-ERK signaling in mosquito cells and on parasite infection [20,21]. In addition to proof-of-principle, the interruption of MEK-ERK signaling via engineered D-site mutations can be translated for the development of transgenic *A. gambiae* that are resistant to malaria parasite development and transmission.

## Competing interests

The authors declare that they have no competing interests.

## Authors’ contributions

AAB and SL conceived and designed the experiments of the study. MC and ACB performed mutagenesis to generate the SNP-encoding *MEK* plasmids. AAB carried out the experiments *in vitro* and KC carried out the experiments *in vivo*. SL and LS conducted statistical analyses. SL and LS prepared the manuscript with AAB. All authors read and approved the final version of the manuscript.
